# Supplementary material for: A Comparative Pharmacokinetic Study of Fexuprazan 10 mg: Demonstrating Bioequivalence with the Reference Formulation and Evaluating Steady State
Source: Pharmaceuticals (Basel). 2023 Aug 11;16(8):1141. doi: 10.3390/ph16081141 (PMC10458111; doi:10.3390/ph16081141)
Supplement: Supplementary file 1 [file pharmaceuticals-16-01141-s001.zip › pharmaceuticals-2541784-supplementary.pdf]

Table S1. Comparison between a newly developed fexuprazan 10mg and the formulation used in first in-human study.

	fexuprazan 10 mg<br>used in this study	fexuprazan 10 mg<br>used in the first in-human study
indices of the size	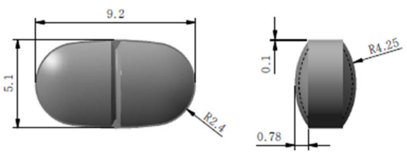	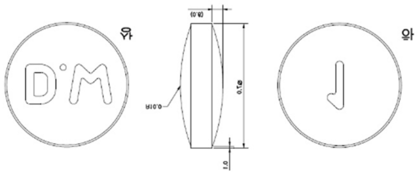
appearance	Orange, oblong, film-coated tablet	White, round, film-coated tablet
length	9.2 mm	7.0 mm
width	5.1 mm	7.0 mm
depth	2.4 mm	3.2 mm
length + width + depth	16.7 mm	17.2 mm
weight	157.5 mg	156 mg
ingredient	fexuprazan 10 mg	fexuprazan 10 mg
